# Endogenous annexin A1 counter-regulates bleomycin-induced lung fibrosis

**DOI:** 10.1186/1471-2172-12-59

**Published:** 2011-10-19

**Authors:** Amílcar S Damazo, André LF Sampaio, Cintia MAG Nakata, Roderick J Flower, Mauro Perretti, Sonia M Oliani

**Affiliations:** 1 Department of Biology; Instituto de Biociências, Letras e Ciências Exatas; São Paulo State University (UNESP), 15054-000, São José do Rio Preto, SP, Brazil; 2 William Harvey Research Institute; Barts and The London School of Medicine and Dentistry, Queen Mary University of London, London EC1M 6BQ, UK; 3 Department of Basic Science in Health, Faculty of Medicine, Federal University of Mato Grosso, 78060-900, Cuiabá, MT, Brazil; 4 Department of Applied Pharmacology; FarManguinhos - FIOCRUZ, 21041-250, Rio de Janeiro, RJ, Brazil

**Keywords:** anti-inflammation, fibrosis, lung inflammation, macrophage, neutrophil, transforming growth factor (TGF-β)

## Abstract

**Background:**

The balancing functions of pro/anti-inflammatory mediators of the complex innate responses have been investigated in a variety of experimental inflammatory settings. Annexin-A1 (AnxA1) is one mediator of endogenous anti-inflammation, affording regulation of leukocyte trafficking and activation in many contexts, yet its role in lung pathologies has been scarcely investigated, despite being highly expressed in lung cells. Here we have applied the bleomycin lung fibrosis model to AnxA1 null mice over a 21-day time-course, to monitor potential impact of this mediator on the control of the inflammatory and fibrotic phases.

**Results:**

Analyses in wild-type mice revealed strict spatial and temporal regulation of the Anxa1 gene, e.g. up-regulation in epithelial cells and infiltrated granulocytes at day 7, followed by augmented protein levels in alveolar macrophages by day 21. Absence of AnxA1 caused increases in: i) the degree of inflammation at day 7; and ii) indexes of fibrosis (assessed by deposition of hydroxyproline in the lung) at day 7 and 21. These alterations in AnxA1 null mice were paralleled by augmented TGF-β1, IFN-γ and TNF-α generation compared to wild-type mice. Finally, treatment of wild type animals with an AnxA1 peptido-mimetic, given prophylactically (from day 0 to 21) or therapeutically (from day 14 onward), ameliorated both signs of inflammation and fibrosis.

**Conclusion:**

Collectively these data reveal a pathophysiological relevance for endogenous AnxA1 in lung inflammation and, more importantly, fibrosis, and may open new insights for the pharmacological treatment of lung fibrosis.

## Background

Pulmonary fibrosis, a severe pathological outcome associated with several lung diseases, can be commonly reproduced by intratracheal instillation of bleomycin, a cytotoxic chemotherapeutic agent. The tissue remodeling that ensues is characterized by severe inflammation (evident from edema and leukocyte migration) and a delayed phase with fibroblast proliferation and excess matrix deposition [1]. The pathological events leading to pulmonary fibrosis have been attributed to an overproduction of interstitial collagens by cytokine-activated fibroblasts [2]; moreover, though a variety of cytokines have been implicated in fibroblast activation, a paramount causative role for transforming growth factor (TGF)-β1 has emerged. This cytokine activates fibroblast differentiation into myofibroblasts [3] and stimulates extracellular matrix (ECM) production [4]. However, besides its pro-fibrotic properties, TGF-β1 exerts a number of other homeostatic functions in immune and cancer biology [5], so that inhibition of TGF-β1 would provoke a series of adverse effects, making it not that valuable as a therapeutic approach. Other therapeutic interventions include anti-inflammatory drugs (e.g. glucocorticoids as prednisone) that are effective to relieve disease without halting fibrosis progression. Anti-fibrotic drugs do not improve lung function or life expectancy and their use may also be associated with harmful side effects [6]. Nonetheless, our knowledge on the underlying mechanisms of pulmonary fibrosis is increased, and this might help for the identification of targets amenable for the development of novel therapies [7].

There is great interest on biochemical pathways centered on endogenous inhibitors endowed with counter-regulatory and protective functions [8]. Most of these studies have focused on acute inflammation, elucidating endogenous anti-inflammatory pathways that operate in parallel, and sometimes in a time-delayed fashion, to the more widely studied pro-inflammatory mediators, to ensure rapid resolution of the host response with return to tissue homeostasis [9]. One line of research has focused on the glucocorticoid-regulated protein annexin A1 (AnxA1; 346 amino acids long; 37 kDa protein), a potent modulator of leukocyte trafficking/transmigration in acute and chronic inflammation [10,11], with a particular ability to inhibit the leukocyte/endothelium interaction in the microvasculature [12]. Characterization of an AnxA1 null mouse colony has revealed, upon stimulation, a dysregulation of pathophysiological mechanisms, with an exacerbation of acute and chronic experimental inflammatory responses [12-15].

The AnxA1 protein is highly expressed in the airways, both in human/animal alveolar macrophages and epithelial cells [16-18], a finding explained by constitutive gene promoter activity in bronchial epithelium and lung endothelial cells [12,15]. The AnxA1 anti-inflammatory effects can be replicated by a shorter peptide spanning the first 25 amino acids, termed peptide Ac2-26. Both AnxA1 and its N-terminal derived peptides exert potent regulation of the inflammatory reaction by activating its receptor, the formyl-peptide receptor (FPR) [19-21], with consequent inhibition of white blood cell trafficking and promotion of efferocytosis [22,23]. Of interest in the context of lung inflammation, down-regulation of endogenous AnxA1 expression has been noted in the bronchoalveolar lavage (BAL) fluid of cystic fibrosis patients [24]. More recently, blockade of the transmembrane conductance regulator protein (CFTR) led to the release of AnxA1 from human and mouse neutrophils, a finding associated with an altered acute inflammatory reaction [25].

Against this background, the present study was undertaken to compare cellular, tissue and macroscopic alterations observed in the model of bleomycin-induced lung fibrosis in wild type and AnxA1 null mice. Moreover, the pattern of expression of the endogenous protein and gene promoter activity were monitored, and the pharmacological potential of AnxA1 peptido-mimetic was assessed.

## Methods

### Animals

Male C57bl/6 wild type and AnxA1 null mice [14,15] (20-25 g of body weight), maintained on a standard chow pellet diet with tap water *ad libitum*, were used for all experiments. Animals were housed at a density of five animals per cage in a room with controlled lighting (lights on from 8:00 a.m. to 8:00 p.m.) in which the temperature was maintained at 21-23°C. Animal work was performed according to UK Home Office regulations (Guidance on the Operation of Animals, Scientific Procedures Act 1986), Committee of Ethics in Animal Research, FAMERP, SP, Brazil (Protocol n°042407) and along the directives of the European Union.

### Murine Model of Lung Fibrosis

Pulmonary fibrosis was induced by intratracheal (i.t.) administration of bleomycin (Blenoxane, Mead Johnson, Princeton, NJ) suspended in sterile phosphate-buffered saline (PBS) at 0.15 U/Kg. Control mice received saline vehicle only (N = 5 per group). At different time post intratracheal injection (day 0, 7 and 21) mice were anesthetized with ketamine (Dopalen, Vetbrands, Brazil, 60%) and xylazine (Anasedan, Vetbrands, Brazil, 40%) solution (60 μL in total) for blood and BAL fluid collection. In some cases, animals were perfused with 20 mL of PBS to remove blood contents from tissues, and lung were collected and processed as described below. Animal survival was monitored daily up to 21 days post-bleomycin. In case of death, the experiments were repeated to achieve the minimal number of 5 animals per group.

In some cases, mice were treated daily with 1 mg/kg intraperitoneally (i.p.) of peptide Ac2-26 (Ac-AMVSEFLKQAWFIENEEQEYVQTVK; obtained from Tocris, Huissen, The Netherlands) starting immediately after the first dose of bleomycin (bleo+pep; N = 5). Another group of bleomycin-treated mice received the same dose and route of peptide Ac2-26 from day 14 to day 21 post-bleomycin (bleo+pep 14-21 d; N = 5). Control mice were treated daily with Ac2-26 (i.p.), but were i.t. treated with saline only instead of bleomycin. In all cases, animals were sacrificed at day 21 for lung histology and immunohistochemistry, and Western blotting analyses.

### Histopathological analyses

Wild type and AnxA1 null lungs were excised, fixed in 4% paraformaldehyde, 0.1 M sodium phosphate buffer (pH 7.4) for 2 h at 4°C; then, they were fragmented, washed, dehydrated in ethanol, clarified in xylene and embedded in paraplast™ (Sigma, USA). Histological sections were cut (Leica RM2265, Leica, GR), mounted on slides, and stained with Masson's Trichrome. Quantification of leukocyte numbers in tissue samples was performed by the point-counting technique [26] with a high-power objective (×40) and measuring the area of analysis with the software *Axiovision *(Zeiss, GR). Data was reported as cells/mm^2 ^(analyzing at least 10 distinct histological sections per mouse).

### Whole Lung Homogenates and Collagen Assays

Total lung hydroxyproline contents were determined using a well-accepted assay [27]. Briefly, samples of lung homogenate (500 μL) were added to 1 mL of 6 N hydrochloric acid for 8 h at 120°C. To a 5 μL sample of the digested lung, 5 μl of citrate/acetate buffer (5% citric acid, 7.2% sodium acetate, 3.4% sodium hydroxide, and 1.2% glacial acetic acid, pH 6.0) and 100 μL of chloramine-T solution (282 mg of chloramine-T, 2 ml of N-propanol, 2 ml of distilled water, and 16 mL of citrate/acetate buffer) were added. The resulting samples were then incubated at room temperature for 20 min before addition of 100 μl of Ehrlich's solution (Aldrich, Milwaukee, WI), 9.3 mL of N-propanol, and 3.9 mL of 70% perchloric acid. After a further incubation at 65°C samples for 15 min, samples were read at 550 nm in a DU640 spectrophotometer (Beckman, Fullerton, CA); hydroxyproline concentrations were then calculated from standard curve (0-100 μg/mL hydroxyproline).

### Cellular analysis of blood and BAL fluids

Aliquots of blood (20 μL) obtained from the abdominal artery were diluted 1:10 in Turk's solution (0.1% crystal violet in 3% acetic acid) for cell quantification. After semi-excision of the trachea, a plastic cannula was inserted and airspaces were washed with 0.5 mL of 0.9% sodium chloride containing 2.6 mM EDTA with a 1 mL syringe. This operation was repeated twice. BAL was centrifuged (600 g for 10 min, 4°C) and the cell-free supernatants were frozen at -80°C for subsequent cytokine analysis. Cell pellets were re-suspended and an aliquot (190 μL) was diluted 20:1 in Turk's solution (3% crystal violet in 20% acetic acid) for cell quantification.

In both cases, total and differential counting was obtained with a Neubauer chamber using a ×40 objective in the light microscope Axioskop II mot plus (Zeiss, GR). BAL cells were distinguished in polymorphonuclear (PMN), mononuclear phagocytic cells (monocyte/macrophages - MPC) and lymphocytes (LY), whereas blood cells were divided into PMN, peripheral blood monocytes (PBMC) and LY.

### Cytokine levels

Aliquots of blood and BAL fluids were centrifuged at 4000 and 600 *g *for 10 min, respectively. Concentrations of tumor necrosis factor (TNF)-α, transforming growing factor (TGF)-β and interferon (IFN)-γ were measured using specific enzyme-linked immunosorbent assay kits purchased from R&D System (Abingdon, UK).

### Immunohistochemistry

AnxA1 protein expression in tissue cells was determined by immunohistochemistry, as reported [13]. Briefly, wild type lung samples were fixed in 4% paraformaldehyde (in 0.1 M sodium phosphate buffer, 2 h at 4°C, pH 7.4) and embedded in LR Gold resin (London Resin, UK). Histological sections (1 μm thick) were blocked with 10% albumin bovine in PBS (PBSA), and a polyclonal rabbit anti-AnxA1 antibody (Zymed Laboratories) added (1:200 in 1% PBSA) overnight at 4°C. As negative control of the reaction, some histological sections of wild type lungs were incubated with non-immune rabbit serum (1:200 working dilution; Sigma-Aldrich) instead of the primary antibody. A goat anti-rabbit IgG (Fc fragment-specific) antibody conjugated to 5 nm colloidal gold (1:100; British BioCell International) was then added. Silver enhancing solution (British BioCell International) was used to augment gold particle staining and histological sections were counterstained with haematoxylin. Analysis of AnxA1 expression was conducted with an Axioskop II microscope (Zeiss, Germany). The stained area was analyzed densitometricaly with the Software *Axiovision*™ (Zeiss) to determine the values of AnxA1 in the cell. The generate values varied from 0 to 255 arbitrary units.

### Anxa1 gene expression by LacZ analysis

Spatial and temporal activation of *Anxa1 *gene promoter in lung was monitored with histochemical X-Gal staining, as reported [12], monitoring Prussian blue precipitation as a marker of LacZ gene activity [14,15]. Briefly, tissues were fixed in 4% paraformaldehyde, 0.1 M sodium phosphate buffer, pH 7.4, for 2 h at 4°C, and washed in rinse buffer (0.1 M phosphate buffer pH 8, 2 mM magnesium chloride, 0.1% Triton X-100). Samples were stained overnight at 37°C with 5 mM potassium ferricyanide and 5 mM potassium ferrocyanide in rinse buffer containing 1 mg/mL 5-Bromo-4-chloro-3-indolyl β-D-galactopyranoside and 4% dimethylformamide. Samples were then embedded in LR Gold™ resin for subsequent microscopy analysis. As a negative control for the reaction, wild type lungs were incubated with X-Gal staining. Densitometric analysis for X-Gal staining was performed densitometricaly with the Software *Axiovision*™ (Zeiss) in lung leukocytes and epithelial cells. The values generated varied from 0 to 255 arbitrary units.

### Western blotting

Lung from control or experimental groups of wild type mice were homogenized in EDTA free protease inhibitor (Roche, UK). Protein concentrations were determined by the Bradford assay (Sigma), mixed 1:1 with Laemmli sample buffer [28], and equal protein amounts (30 μg) electrophoresed in a 10% polyacrylamide gel in running buffer (0.3% Tris base, 1.44% glycine, 0.1% SDS in distilled water) followed by transfer of proteins onto Hybond-C extra nitrocellulose membranes in transfer buffer (0.3% Tris base, 1.44% glycine, 20% methanol in distilled water). After membrane blocking with 5% non-fat milk solution in TBS containing 0.1% Tween 20, AnxA1 immunoreactivity was assessed using specific antibody to detect both cleaved (33 kDa) and intact protein (37 kDa) (1:1000; Invitrogen, USA). The signal was amplified with HRP-linked anti-mouse secondary antibody (1:2000; Amersham Biosciences, USA) and visualized by ECL (Western blotting detection reagent; Amersham Biosciences, USA).

### Data handing and statistical analysis

In all cases data are reported as mean ± SEM of five mice per group. Statistical differences between groups were determined by ANOVA followed, if significant, by the Bonferroni test. In all cases, a probability value <0.05 was taken as significant. The survival rate was analyzed using Kaplan-Meier cumulative plots, and comparisons between groups were performed using a log-rank test.

## Results

### Analysis of AnxA1 protein expression and gene promoter activity in lung fibrosis

We began this study by monitoring AnxA1 protein expression in wild type and gene promoter activity in AnxA1 null mice, both in bronchiolar epithelial cells, circulating and migrated leukocytes. Table 1 and 2 reports the cumulative data obtained from these densitometric analyses.

**Table 1:** Annexin-A1 protein expression in wild type mouse lung tissue and leukocytes during the process of fibrosis induced by bleomycin

Wild type	Epithelial cells	PMN			MPC		
	
		Intra-vascular	Trans-migrated	Intra-alveolar	Intra-vascular	Tissue	Intra-alveolar
**Sham**	45 ± 2	101 ± 7	62 ± 2	80 ± 8	41 ± 3	43 ± 4	72 ± 4
**Bleo 7d**	66 ± 3 *	63 ± 3 *	83 ± 2 *	82 ± 7	63 ± 2 *	72 ± 4 *	74 ± 5
**Bleo 7d + Ac2-26**	70 ± 5 *	62 ± 2 *	62 ± 3 #	73 ± 8	45 ± 3 #	74 ± 5 *	73 ± 4
**Bleo 21d**	48 ± 2	64 ± 3 *	70 ± 4	70 ± 7	42 ± 1	44 ± 4	43 ± 4 *
**Bleo 21d + Ac2-26 daily**	60 ± 3 *#	110 ± 9 #	86 ± 5 *	79 ± 8	55 ± 3	44 ± 3	104 ± 6 #
**Bleo 21d + Ac2-26 14-21d**	70 ± 4 *#	70 ± 3 *	91 ± 7 *#	81 ± 7	45 ± 4	50 ± 2	55 ± 1

AnxA1 protein expression was analyzed, as described in Material and Methods, in wild type mice lung at 0, 7 and 21 days post-bleomycin i.t. administration. Also a group of mice were daily i.p. treated or post-treated with the peptide Ac2-26 as described in Material and Methods section. Values (densitometric units) are mean ± SEM of 10 histological tissue sections analyzed from five mice per group. * P < 0.05 versus sham group values. # P < 0.05 versus corresponding control group values. PMN, polymorphonuclear cells; MPC, monocyte/macrophages.

**Table 2:** Densitometric analysis of LacZ gene expression in specific lung cells during the process of fibrosis induced by bleomycin

Epithelial cells	PMN			Mono	MPC		
	
		Intra-vascular	Trans-migrated	Intra-alveolar	Intra-vascular	Tissue	Intra-alveolar
**Sham**	61 ± 3	53 ± 2	60 ± 2	65 ± 2	50 ± 3	55 ± 2	65 ± 3
**Bleo 7d**	108 ± 10 *	65 ± 3	103 ± 5 *	110 ± 6 *	63 ± 2	70 ± 3 *	73 ± 3
**Bleo 21d**	180 ± 20***	60 ± 3	105 ± 4*	108 ± 5*	61 ± 2	105 ± 5 **	111 ± 10 *

AnxA1 null mice were treated with bleomycin (0.15 U/Kg i.t.) at time 0 and lung collected at the reported time points. X-Gal staining was done as described in Material and Methods. Values (densitometric units) are mean ± SEM of 10 histological tissue sections analyzed from five mice per group. * P < 0.05 versus sham group values. ** P < 0.01 versus sham group values. *** P < 0.001 versus sham group values.

**Figure 1:**
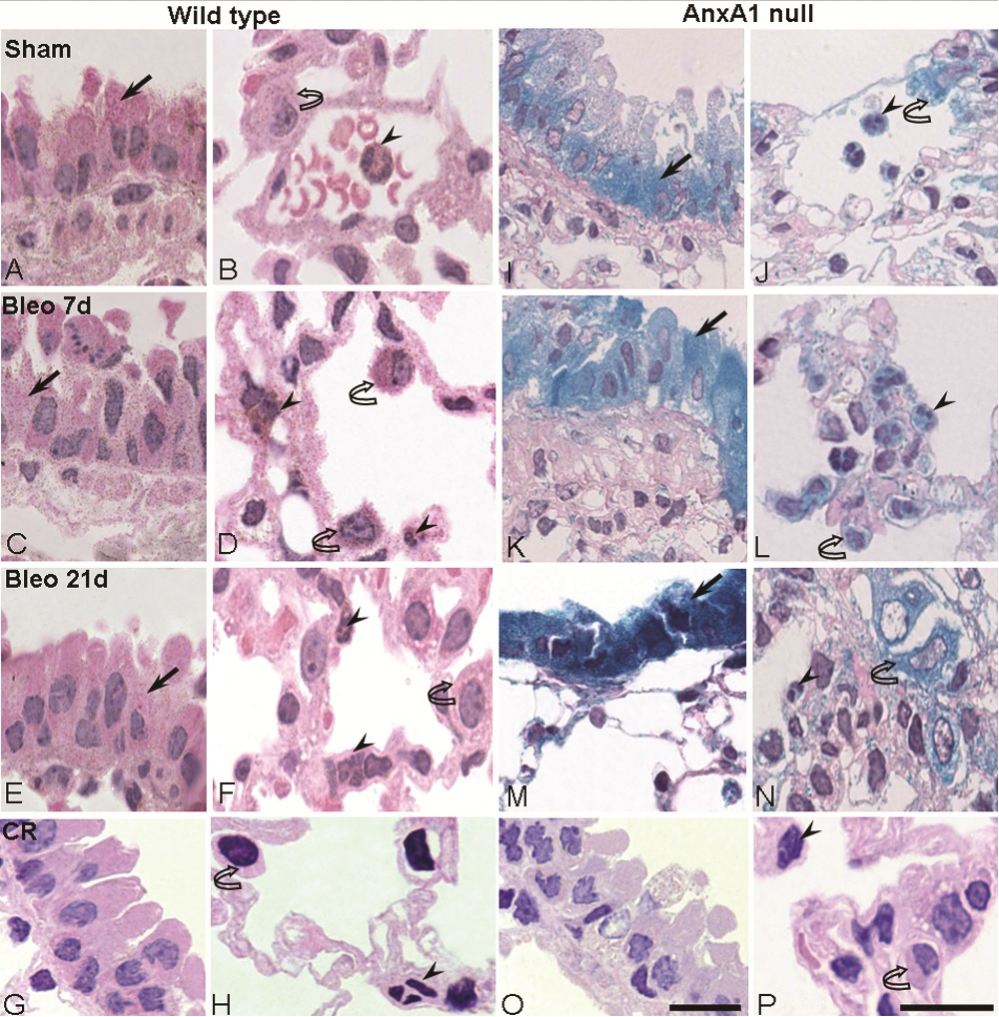
**Analysis of endogenous AnxA1 protein and *Anxa1 *gene expression during lung fibrosis**. (A-H;O-P) Wild type and (I-N) AnxA1 null mice received bleomycin *i.t. *at time 0. The AnxA1 protein content was analyzed by immunohistochemistry. At 0 time-point, wild type mice exhibit a basal immunostain for AnxA1 protein (A and B). After 7 days post-bleomycin administration, this protein expression was greatly increased (C and D). And, after 21 days, the AnxA1 expression was reduced (E and F) in epithelial cells (arrow) and polymorphonuclear (PMN) (arrowheads) and MPC (curve arrow)(G and H). Control of immunogold reaction (CR) showing no cellular immunostaining. *Anxa1 *gene promoter activity was visualized by X-Gal staining reaction. AnxA1 null mice lung showing *Anxa1 *gene expression (I and J) on the epithelial cells (arrow), intravascular PMN (arrowhead) and MPC (curve arrow); an intense positive reaction was attained (K-N) on day 7 and 21 post-bleomycin administration. Control for LacZ reaction (CR) showing wild type mice negative to the X-Gal staining (O and P). Haematoxylin counterstain. Bars, 10 μm.

As predicted [12], immunohistochemistry revealed intracellular localization for AnxA1 associated to gold particles in bronchiole epithelial cells (Figure 1A), mononuclear phagocytic cell (MPC) and circulating polymorphonuclear (PMN) (Figure 1B). All cells were positive for AnxA1 under basal conditions (Table 1). At day 7 post-bleomycin, an increment of AnxA1 expression was observed in the bronchiolar epithelium (Figure 1C), PMN and MPC of wild type mice (Figure 1D, with quantitative data shown in Table 1); this cellular response was no longer evident at day 21 (Figure 1E and 1F, Table 1). No immunogold staining could be detected in absence of the primary antibody (Figure 1G and 1H), confirming the specificity of the AnxA1 staining.

In lungs taken from sham mice (day 0), basal *Anxa1 *gene promoter activity could be detected in the bronchiolar epithelium (Figure 1I), PMN and MPC (Figure 1J). This colorimetric reaction was markedly augmented at day 7 (Figure 1K and L) and day 21 (Figure 1M and 1N) post-bleomycin, with the epithelium and MPC resulting strongly positive at the later time-point. Table 2 reports these data in quantitative fashion. As a control for these data, performing the X-Gal staining protocols on lungs taken from wild type mice did not yield any coloration (Figure 1O and 1P).

### Lack of AnxA1 exacerbates inflammatory cell activation in lung fibrosis

**Figure 2:**
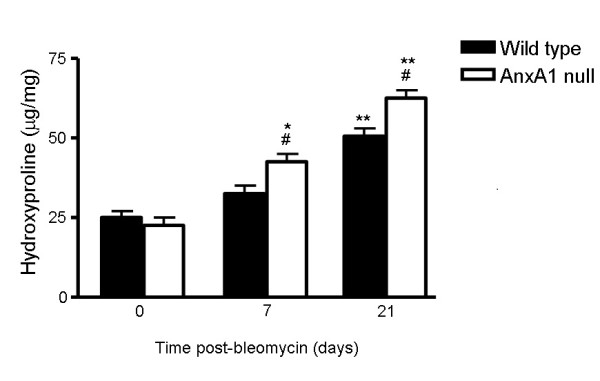
**Leukocyte influx into the lung tissue**. Semiquantitative analysis of the histological sections showing intravascular polymorphonuclear (PMN) and monocytes (mono), and transmigrated PMN and MPC in lung tissue. Wild type and AnxA1 null mice were analyzed at 0, 7 and 21 days post-bleomycin *i.t. *administration as described in Methods section. Data are mean ± SEM from 5 mice per time point. *P < 0.05 versus 0 time point wild type group values; **P < 0.01 versus 0 time point wild type group values; ***P < 0.001 versus 0 time point wild type group values; #P < 0.05 versus corresponding wild type group values; ###P < 0.01 versus corresponding wild type group values.

The early response to bleomycin-induced fibrosis is characterized by a dramatic increase in inflammatory cell recruitment that precedes fibrotic tissue formation [7]. Bleomycin administration induced marked leukocyte influx in wild type mice with no influx of blood cells being found in untreated mice (Figure 2). Quantitative analysis of several histological lung sections of AnxA1 null and wild type mice revealed rapid and intense leukocyte migration not only in peribronchial and perivascular areas but also in the interstitial space, occurring both at day 7 and 21 post-bleomycin (Figure 2). At day 7, mice deficient in AnxA1 displayed a higher degree of PMN and peripheral blood monocytes (PBMC) adhesion to lung microvessels (Figure 2).

Analysis of several tissue parameters was complemented by quantification of blood and BAL total leukocyte numbers. In wild type mice, bleomycin administration caused marked leukocytosis at day 21, with a high number of circulating PMN, PBMN and lymphocytes (LY) being calculated (Additional file 1: Figure S1). These profiles of circulating leukocyte subsets were mildly affected by absence of AnxA1: a reduction in LY at day 7 as well as a less pronounced increase in PMN could be measured at day 21 (Additional file 1: Figure S1). In parallel analyses, a rapid cells influx of blood-borne leukocyte into BAL fluid was detected, with marked changes between wild type and AnxA1 null mice especially for PMN (day 7 and day 21), PBMC (day 21) and LY (day 7) (Additional file 1: Figure S1).

### Histopathological and biochemical analysis of the lung

**Figure 3:**
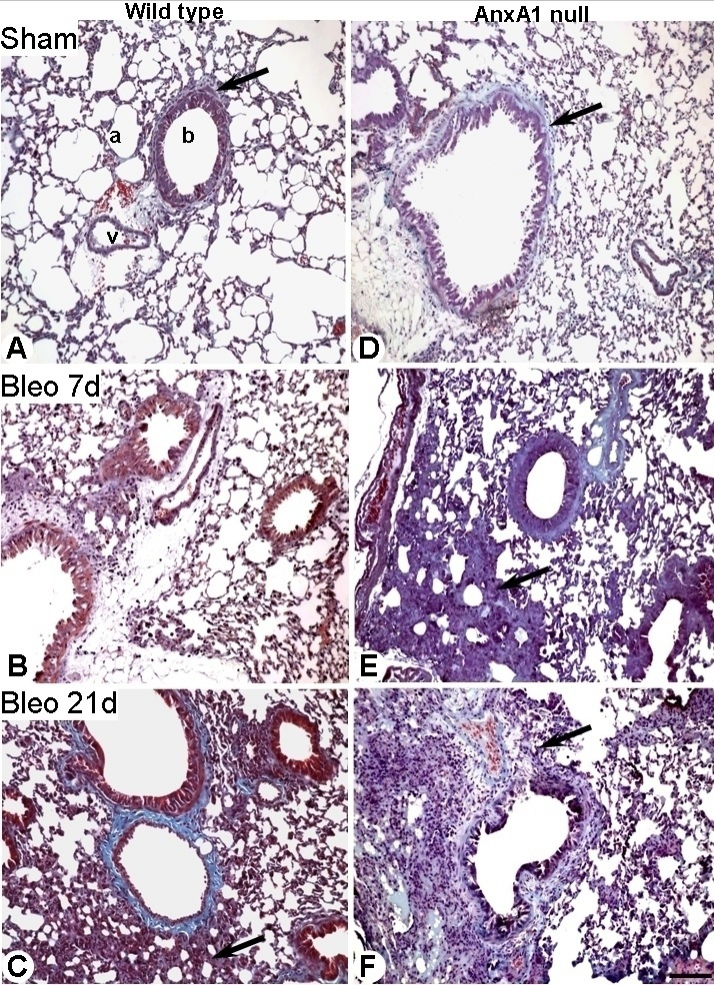
**Lung histopathology**. (A-C) Wild type and (D-F) AnxA1 null mice lung were analyzed at 0, 7 and 21 days post-bleomycin i.t. administration as described in Material and Methods section. (A and D) Histological analysis of wild type and AnxA1 null mouse lungs showing presence of collagen in the connective tissue in the lung parenchyma (a), near the vessel (v) and bronchiole (b). (B) No major changes in the connective tissue of wild type lung parenchyma, as observed at day 7 post-bleomycin administration. (C) At day 21 post-bleomycin, the alveolar septa thicken because of a significant increase in connective tissue deposit (arrow). (E and F) In the AnxA1 null mice, the fibrosis was evident already at day 7; by day 21 there is evidence of increased connective tissue (arrows). Masson trichrome stain. Bars, 100 μm.

We then assessed the extent of pulmonary fibrosis in both genotypes, using the Masson tri-chrome stain. Administration of saline to either wild type or AnxA1 null did not lead to abnormal deposition of collagen around the bronchiole, vessels or alveoli (Figure 3A and 3D). In contrast, at day 7 post-bleomycin, wild type mice showed no significant increase in the connective tissue (Figure 3B), with signs of severe fibrosis evident at day 21, with greater destruction of alveolar architecture, more pronounced at perivascular and peribronchial sites (Figure 3C). These changes were also evident in AnxA1 null mice, both at 7 and 21 days post-bleomycin (Figures 3E and 3F).

Results obtained with the histopathological method were confirmed by measuring hydroxyproline content in lung tissue samples, to quantify the extent of collagen deposition. Both wild type and AnxA1 null bleomycin-treated mice had significantly higher fibrosis scores than saline-treated corresponding mice (Additional file 2: Figure S2). Moreover, there was a statistically significant difference (+23.5% at day 7; +20% at day 21) between wild type and AnxA1 null bleomycin-treated mice, indicating induction of a higher degree of pulmonary fibrosis in the latter genotype (Additional file 2: Figure S2).

### Bleomycin-induced fibrosis is lethal in AnxA1 null mice

**Figure 4:**
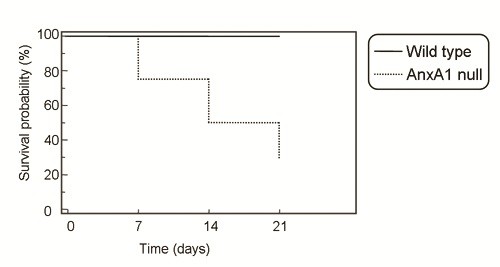
**Bleomycin lethality in AnxA1 null mice**. Wild type and AnxA1 null mice received 0.15 U/Kg bleomycin i.t. at time 0 and survival rate was monitored daily up to 21 days. Results are cumulated from three experiments with a total of 15 mice per group.

Why the observation is surprising to observe that a proportion of AnxA1 null mice succumbed during the time-course of these experiments, with a progressive incidence of lethality from day 7: at day 21 ~50% of bleomycin-treated mice had died (Figure 4). No lethality was observed in wild type animals up to the day 21.

### Cytokine levels in bleomycin-induced fibrosis

It is accepted that TGF-β1, IFN-γ and TNF-α are key cytokines in the fibrotic process of the lung initiated by bleomycin [3,29]. Thus, we analyzed cytokine levels as a possible cause behind the increased signs of fibrosis and collagen accumulation detected in AnxA1 null mice.

**Figure 5:**
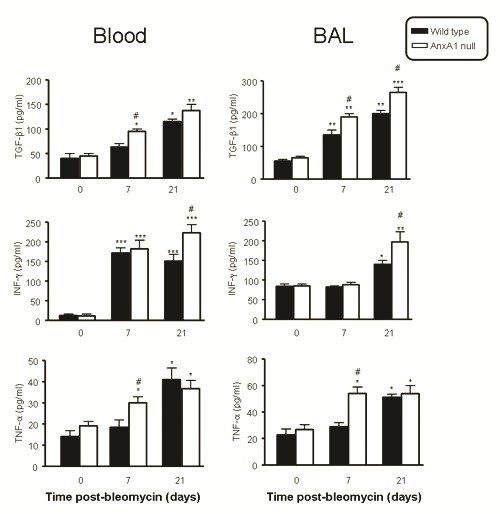
**Serum and BAL supernatant cytokine levels**. Wild type and AnxA1 null mice received bleomycin i.t. at time 0, with blood and BAL samples being collected at the reported time points. The serum and cell-free supernatant BAL concentration of TGF-β1, IFN-γ and TNF-α was determined by ELISA. Results are mean ± SEM from two separate experiments with 5 mice per group. *P < 0.05,**P < 0.01 and ***P < 0.001 versus 0 time point wild type group values; #P 0.05 versus corresponding wild type group values.

Figure 5 shows a marked time-dependent increase (>15-fold) in circulating IFN-γ upon bleomycin treatment, whereas changes in TGF-β1 and TNF-α were more modest (between 2-3 fold over untreated mice, day 0 values). Furthermore, higher serum TGF-β1 and TNF-α levels were measured at day 7 in AnxA1 null mice. Similar changes were seen in BAL fluid samples, with discrete alterations of these values in samples taken from AnxA1 null mice (Figure 5). As an example, BAL TNF-α was higher at day 7, with BAL IFN-γ being increased at day 21. TGF-β1 was mildly affected by the absence of endogenous AnxA1.

### Regulatory actions of peptide Ac2-26

Finally, we tested whether treatment of wild type mice with an AnxA1 mimetic would affect the dramatic changes provoked by bleomycin in the lung. Two protocols were used for administration of this peptide.

**Figure 6:**
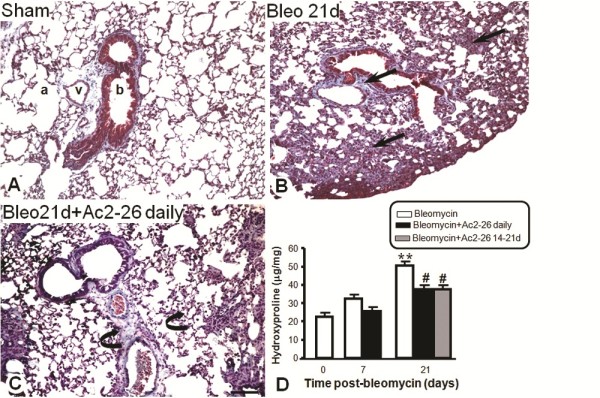
**Effects of treatment with peptide Ac2-26 on lung fibrosis and hydroxyproline content**. Lung were analyzed at 0 and 21 days post-bleomycin i.t. administration. Also a group of mice were daily treated i.p. with the peptide Ac2-26 (1 mg/kg) or between the days 14 to 21 post-bleomycin as described in Methods section. (A) Morphological analysis of sham group indicated the reduced content of collagen in the lung parenchyma (a), near the vessels (v) and bronchiole (b). (B) At day 21 after bleomycin administration, the alveolar septa thickening indicated the significantly increasement of collagen deposit (arrows). (C) The daily treatment with peptide Ac2-26 prevents bleomycin-induced lung fibrosis (curve arrows). Masson trichrome stain. Bar, 100 μm. (D) Pulmonary fibrosis was biochemically assessed by measurement of lung hydroxyproline content. Results are expressed as means ± SEM of μg of hydroxyproline per mg of lung tissue. **P < 0.01 versus 0 time point values; #P < 0.05 versus day 21 post-bleomycin time point values.

Histological analyses showed that the peptide Ac2-26 treatment reduced the extracellular deposition of collagen when compared to mice treated with bleomycin alone (Figure 6B and 6C). In the group of mice treated with peptide Ac2-26, daily from day 0, a clear reduction in connective tissue deposition around the bronchiolar epithelium was observed, with some similarity to the morphological structure of the sham group (Figure 6A and 6C). Similar protection of the lung was afforded when the peptide was administered between days 14 to 21 post-bleomycin, as determined by quantification of lung hydroxyproline content (Figure 6D). A control group treated daily with peptide Ac2-26 i.p., and given saline i.t., did not show significant morphological changes in the lung tissue (data not shown).

**Figure 7:**
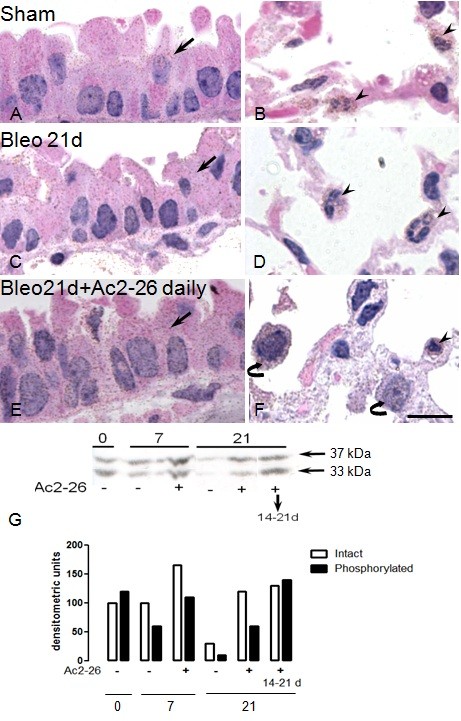
**Analysis of endogenous AnxA1 expression in wild type mice after treatment with peptide Ac2-26**. Mice received bleomycin *i.t. *at time 0. Immunohistochemistry was performed to visualize the protein expression in epithelial cells (arrows), PMN (arrowheads) and alveolar macrophages (curved arrow). (A and B) Sham mice exhibit a basal immunostain for AnxA1 protein. (C and D) Lung fibrosis induced by bleomycin reduced the AnxA1 expression in epithelial cells (arrow) and PMN (arrowheads). (E and F) Daily treatment with peptide Ac2-26 (1 mg/kg) increased the AnxA1 expression during lung fibrosis. Haematoxylin counterstain. Bar, 10 μm. (G) The AnxA1 protein content was also analyzed by Western blotting (seen as both the native 37 kDa and 33 kDa cleavage products). AnxA1 was down regulated during lung fibrosis. Daily i.p. treatment with peptide Ac2-26, or given only between day 14 and day 21 post-bleomycin, increased expression of this endogenous protein.

Treatment of mice with peptide Ac2-26 led to an increased expression of endogenous AnxA1 protein in the lung (Figure 7E and 7F), when compared to bleomycin treatment alone (Figure 7C and 7D). The changes provoked by peptide Ac2-26 occurred in the alveolar macrophage and the epithelial cell and the expressions were higher than sham group (saline treated) (Figure 7E/F and 7A/B, respectively). Western blotting analyses confirmed these histological results, showing augmented AnxA1 in lung protein extracts prepared from mice treated with peptide Ac2-26 over 21 days or only for the 14-21 day period, when compared to bleomycin-treated control mice (Figure 7G). Table 1 reports quantitative data associated with these analyses at single cell level.

## Discussion

Pulmonary fibrosis has a complex etiology involving interactions among multiple cell types and intricate cytokine/chemokine networks. However, the precise role of any one of these cytokines *in vivo *is hard to identify in view of their pleiotropic biological activities. Studies using animal models afford a well-defined means of addressing this problem since provide the opportunity to target each of these putative mediators using immunological and genetic approaches.

In line with the human fibrosis, the mechanisms underlying bleomycin-induced lung fibrosis are not fully understood, although several studies have identified expression of specific cytokines and mediators with potential functional role(s) during this process. We propose here that investigation of the potential modulation of effectors of endogenous anti-inflammation could provide novel clues to harness the host response during lung fibrosis [30]. Such a novel approach - exploiting the functions of homeostatic and tissue protective mediators - would possibly be more successful than blockade of the actions of pro-inflammatory mediators since anti-cytokine therapy has produced no improvement in lung function and, rather, seemed to be associated with harmful side effects [6]. Few studies in humans and animals have demonstrated down regulation of one endogenous effector of resolution, the protein AnxA1, during the development of cystic fibrosis [24,25]; the present study was intended to address, for the first time, the potential relevance of AnxA1 in experimental fibrosis using the recently generated AnxA1 null mouse [14,15].

Analysis of AnxA1 protein expression in the bleomycin-induced fibrosis indicated increased expression in bronchial epithelial cells and leukocytes during the inflammatory phase (7 days) followed by a reduction during the fibrotic phase (21 days), as compared to sham animals: this event is in agreement with changes reported in lung tissue and leukocytes [24,25] in experimental settings modeling tissue fibrosis.

We could monitor *Anxa1 *gene promoter activity in leukocytes and epithelial cells using the AnxA1 null mouse colony [12] where a LacZ gene reporter assay is inserted in frame. At day 21 post-bleomycin, the X-Gal staining detected higher *Anxa1 *gene promoter activity in leukocytes and epithelial cells compared to day 0 lung tissue samples. These data, together with the AnxA1 protein expression, indicate that despite the lower levels of AnxA1 detected in the cells, there is ample *Anxa1 *gene promoter activity; we wish to postulate that reduction in AnxA1 protein may be associated with a post-transcriptional mechanism(s) which could perhaps lead to AnxA1 proteolysis or leakage (aberrant secretion) from the cells. AnxA1 lacks a signal peptide yet it is released from cells (e.g. activated PMN; see ref. 31). Several non-canonical secretory pathways have been ascribed, possibly in a cell specific fashion, including phosphorylation [32], proteolysis [24,33], granule/membrane fusion [34] and microparticles release [35]. Future studies will attempt to establish the 'preferred' mode of AnxA1 secretion in activated lung cells, though it cannot be excluded that alternative mechanisms might be operating in diseased cells.

At day 21, we could also measure a higher degree of fibrosis and collagen deposition in AnxA1 null animals, as detected both morphologically and by hydroxyproline measurement. Therefore, AnxA1 down-regulation might be an important determinant during lung fibrosis development, suggesting that manipulation of local pulmonary concentration of AnxA1, perhaps by adenoviral delivery (a route successfully applied to lung pathologies; [36], might represent a novel way to afford lung tissue protection.

AnxA1 has been implicated in many aspects of cell physiology including regulation of cell growth [37] and differentiation [38], signal transduction and arachidonic acid release [39], as well as intracellular vesicle trafficking [40]. Moreover, it promotes cell apoptosis and, possibly more relevant, phagocytosis of apoptotic cells and subsequent tissue clearance [23] has also been ascribed to this homeostatic mediator.

Croxtall et al. [37] showed that a lung fibroblast cell line derived from the AnxA1 null mouse was refractory to growth inhibition by dexamethasone, indicating that intracellular AnxA1 (though it cannot be excluded also an extracellular role here) can function as a negative regulator of cell growth. In pathology, AnxA1 has been implicated directly or indirectly in tumor cell growth [41,42]. Our previous results also showed an abnormal growth formation in the AnxA1 null mice skull, probably caused by increased extracellular matrix and a delay in the bone mineralization [43]. Thus, we would postulate that absence of AnxA1 may lead to higher cell proliferation, increasing the extracellular matrix production and fibrosis formation. Studies by Nusrat's lab [44] have identified AnxA1 as a major effector of tissue protection following epithelial damage, an effect requiring externalization of the protein.

In our experimental conditions, absence of AnxA1 affected several indices of the response promoted by bleomycin, characterized by an early inflammatory phase followed by the fibrotic process. The combined effect of AnxA1 absence and bleomycin-induced fibrosis exceeded the sum of the effects of each factor alone on the severity of histological changes. This was visible because the higher degree of activation of circulating leukocyte and a remarkable leukocyte infiltration to the alveolar space and lung parenchyma; these cellular events led, or at least contribute, to: i) a more severe inflammatory response and ii) a greater destruction of alveolar architecture, pronounced in the perivascular and peribronchial areas. This effect is in line with our previous data in different experimental models of inflammation, including acute peritonitis [13] and lipopolysaccharide-induced systemic inflammation [12]. Absence of AnxA1 is conducive to increased leukocyte infiltration leading to a more severe inflammatory reaction. Several studies have associated inflammatory cell recruitment and lung damage with fibrosis formation [7,45], prompting us to propose that alteration in the early protective role of AnxA1 during the inflammatory phase could lead, downstream, to a higher degree of fibrosis.

So far we have two feasible mechanisms for explaining the impact of AnxA1 in this model: i) direct alteration in the phenotype of lung cells potentially, but not necessarily, related to a ii) more aggressive inflammatory reaction. Analysis of cytokine content favors a functional association between these two readouts. In fact, tightly coupled to the early inflammatory response is a higher cytokine expression. We measured TNF-α, TGF-β1 and IFN-γ since they instrumental for the inflammatory process and/or fibrosis [46,47]. In wild type mice, levels of these cytokines changed in the blood and BAL fluid samples. TGF-β1 is largely responsible for fibroblast activation/differentiation, as well as collagen over-expression [3]. Furthermore, a lower degree of parenchymal inflammation has been observed in IFN-γ deficient mice subjected to the bleomycin-induced lung fibrosis model [45]. In our samples we could determine a correlation between increased levels of TGF-β1 with TNF-α and IFN-γ up-regulation. Hardie and collaborators [48] showed that TGF-β1 conditional over-expression in epithelial cells, endothelial cells and macrophages induces fibrosis and TNF-α expression even in the absence of inflammation. It is plausible that complex positive loops might be activated locally, since TGF-β1 induction from macrophages occurs in a two-stage process requiring both TNF-α and IL-13 [49].

Global absence of AnxA1 resulted in dysregulated pulmonary levels of TNF-α, TGF-β1 and IFN-γ, more evident at day 21, yet present also at day 7 post-bleomycin. This association of AnxA1 with TNF-α expression is not totally unexpected as previously reported in a model of systemic endotoxemia induced by LPS [12,50]. More studies will better address the molecular events behind AnxA1 regulation of cytokine production in lung cells.

Finally, we tested the potential pharmacological benefit of treatment of mice with peptide Ac2-26. This peptide mimics most [10,31,51], if not all [21], of the pharmacological actions of AnxA1. Given daily over 21 days, or from day 14 to 21, peptide Ac2-26 reduced the most remarkable feature of the animal response to bleomycin, which is fibrosis, as quantified both histologically and by determination of lung hydroxyproline content.

An interesting observation stemmed from these experiments, that is the positive effect of treatment of mice with peptide Ac2-26 on tissue levels of AnxA1, indicating that a positive loop might exist in anti-inflammation, whereby AnxA1 activation of its receptor (here the activation is achieved by the AnxA1 pharmacophore peptide Ac2-26) would augment its own synthesis, in epithelial cells and in some leukocytes subsets (Table 1). Such a model is congruent with a recent study in mouse colitis, which revealed the ability of treatment with peptide Ac2-26 to increase tissue mRNA for AnxA1 receptors [44]. The same held true for epithelial cells treated with AnxA1 [52]. Therefore, different studies are coming together with indications for the existence of this positive loop; if extended to other settings; this would indicate possible "auto-reactivity" of endogenous anti-inflammation and resolution, at least with respect to the AnxA1 pathway.

## Conclusions

In summary, the striking phenotype we describe here indicates that endogenous and exogenous AnxA1 affects selective aspects of the process of fibrosis development in the lung, a feature that may warrant further investigation on AnxA1 biology in these experimental and pathological settings.

## Abbreviations

AnxA1: annexin A1; BAL: bronchoalveolar lavage; Bleo: bleomycin; ECM: extracellular matrix; IFN-γ: interferon-γ; LY: lymphocytes; MPC: mononuclear phagocytic cells; PBMC: peripheral blood monocytes; PBS: phosphate-buffered saline; PMN: polymorphonuclear; TGF-β1: transforming growth factor-β1; TNF-α: tumor necrosis factor-α.

## Competing interests

The authors declare that they have no competing interests.

## Authors' contributions

ASD: participated in the design of the study, carried out the experiments and participated in the sequence alignment and drafted the manuscript; ALFS: carried out the experiments; CMAGN: carried out the histopathological analysis; RJF: provided de transgenic mice and helped during the discussion of the manuscript; MP: conceived of the study, and participated in its design and coordination and helped to draft the manuscript; SMO: participated in the design of the study and participated in the sequence alignment and drafted the manuscript.

All authors read and approved the final manuscript.

## Supplementary Material

Additional file 1 **Figure 1S. Time course of leukocyte influx in the blood and the bronco-alveolar lavage (BAL)**. Wild type and AnxA1 null mice received bleomycin i.t. at time 0. At different time points, blood aliquots were collected for lymphocyte (LY), peripheral blood monocyte (PBMN) and polymorphonuclear (PMN) quantification; BAL were also performed for measuring LY, mononuclear phagocytic cells (MPC) and PMN. Data are mean ± SEM from two separate experiments with 5 mice each. *P < 0.05, **P < 0.01 and ***P < 0.001 versus 0 time point wild type group values; #P < 0.05 and ###P < 0.01 versus corresponding wild type group values. Click here for file

Additional file 2 **Figure 2S. Effects of *Anxa1 *gene deletion on lung hydroxyproline content**. Pulmonary fibrosis was biochemically assessed by measurement of lung hydroxyproline content at 0, 7 and 21 days post-bleomycin i.t. administration in the wild type and AnxA1 null mice as described in Material and Methods section. The absence of AnxA1 significantly increased the hydroxyproline content induced by bleomycin. Results were expressed as means ± SEM of μg of hydroxyproline per mg of lung tissue. *P < 0.05 and **P < 0.01 versus 0 time point wild type group values; #P < 0.05 versus corresponding wild type group values. Click here for file
