# Supplementary figures and images for: Endogenous annexin A1 counter-regulates bleomycin-induced lung fibrosis
Source: BMC Immunol. 2011 Oct 19;12:59. doi: 10.1186/1471-2172-12-59 (PMC3212807; doi:10.1186/1471-2172-12-59)

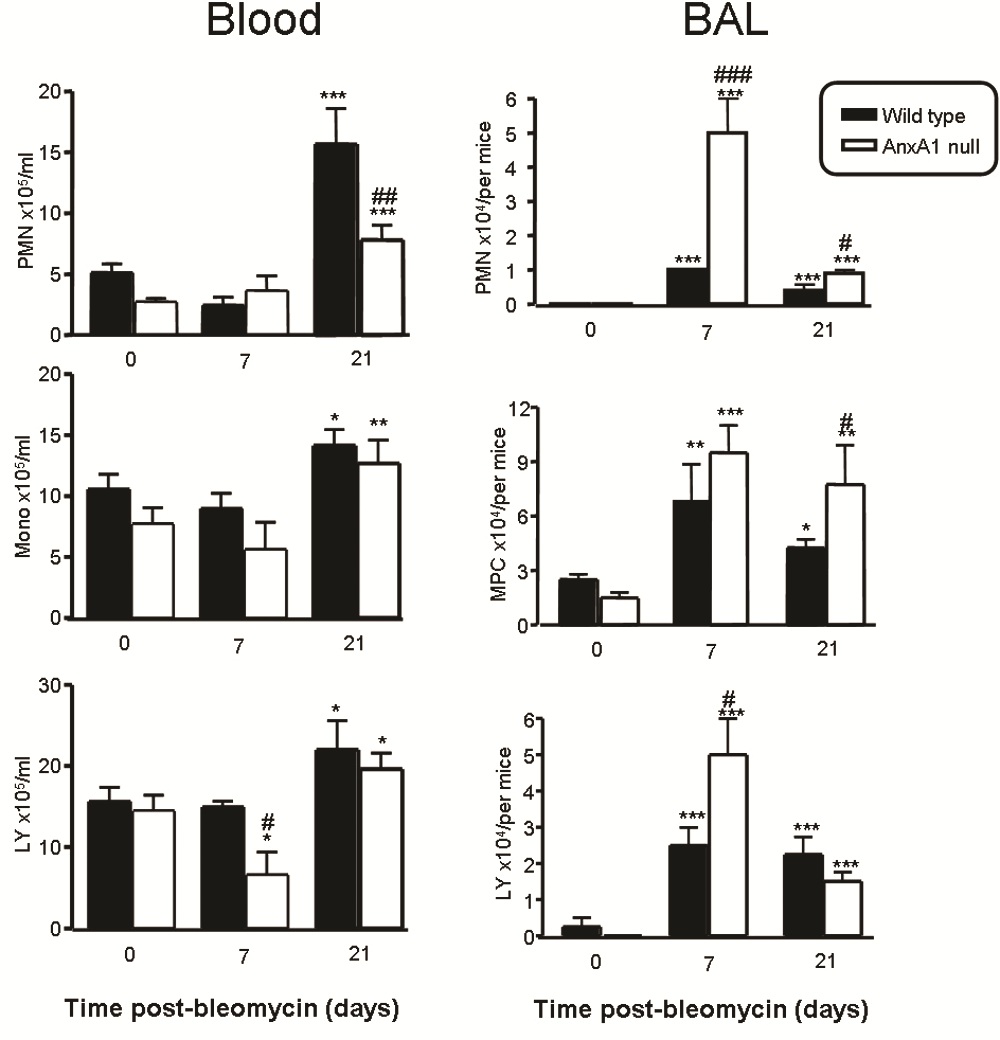

Supplement: Additional file 1 — Figure 1S. Time course of leukocyte influx in the blood and the bronco-alveolar lavage (BAL). Wild type and AnxA1 null mice received bleomycin i.t. at time 0. At different time points, blood aliquots were collected for lymphocyte (LY), peripheral blood monocyte (PBMN) and polymorphonuclear (PMN) quantification; BAL were also performed for measuring LY, mononuclear phagocytic cells (MPC) and PMN. Data are mean ± SEM from two separate experiments with 5 mice each. *P < 0.05, **P < 0.01 and ***P < 0.001 versus 0 time point wild type group values; #P < 0.05 and ###P < 0.01 versus corresponding wild type group values. [file 1471-2172-12-59-S1.JPEG]

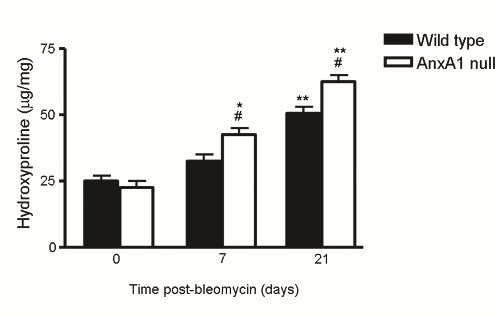

Supplement: Additional file 2 — Figure 2S. Effects of Anxa1 gene deletion on lung hydroxyproline content. Pulmonary fibrosis was biochemically assessed by measurement of lung hydroxyproline content at 0, 7 and 21 days post-bleomycin i.t. administration in the wild type and AnxA1 null mice as described in Material and Methods section. The absence of AnxA1 significantly increased the hydroxyproline content induced by bleomycin. Results were expressed as means ± SEM of μg of hydroxyproline per mg of lung tissue. *P < 0.05 and **P < 0.01 versus 0 time point wild type group values; #P < 0.05 versus corresponding wild type group values. [file 1471-2172-12-59-S2.JPEG]
